# Genes that Control Vaccinia Virus Immunogenicity

**DOI:** 10.32607/actanaturae.10935

**Published:** 2020

**Authors:** S. N. Shchelkunov, G. A. Shchelkunova

**Affiliations:** State Research Center of Virology and Biotechnology “Vector”, Rospotrebnadzor, Novosibirsk region, Koltsovo, 630559 Russia; The Federal Research Center Institute of Cytology and Genetics, Siberian Branch, Russian Academy of Sciences, Novosibirsk, 630090 Russia; Novosibirsk State University, Novosibirsk, 630090 Russia

**Keywords:** smallpox, vaccination, immunogenicity, protectiveness, immune modulating proteins

## Abstract

The live smallpox vaccine was a historical first and highly effective vaccine. However, along with high immunogenicity, the vaccinia virus (VACV) caused serious side effects in vaccinees, sometimes with lethal outcomes. Therefore, after global eradication of smallpox, VACV vaccination was stopped. For this reason, most of the human population worldwide lacks specific immunity against not only smallpox, but also other zoonotic orthopoxviruses. Outbreaks of diseases caused by these viruses have increasingly occurred in humans on different continents. However, use of the classical live VACV vaccine for prevention against these diseases is unacceptable because of potential serious side effects, especially in individuals with suppressed immunity or immunodeficiency (e.g., HIV-infected patients). Therefore, highly attenuated VACV variants that preserve their immunogenicity are needed. This review discusses current ideas about the development of a humoral and cellular immune response to orthopoxvirus infection/vaccination and describes genetic engineering approaches that could be utilized to generate safe and highly immunogenic live VACV vaccines.

## INTRODUCTION

The emergence and development of vaccinology was primarily associated with the search for ways to protect against diseases such as smallpox (Latin *variola*), a particularly dangerous infection that causes epidemics with a mortality rate of up to 40% or more in infected patients. Smallpox survivors were easily identified by the characteristic scars on their face skin (the so-called “pitted face”), which were left on the sites of pustules after the loss of dry crusts; these people became immune to smallpox whenever a new outbreak of the disease occurred. Apparently, these observations formed the basis for the inoculation of infectious material obtained by collecting skin crusts from smallpox patients into skin incisions (usually in the forearm), or intranasally, made on healthy people in India and China. This procedure, called *variolation* (from variola inoculation), caused a moderately severe disease and provided further reliable protection against smallpox. However, 0.5 to 2% of variolated patients would die, which prevented widespread use of this procedure [1].

In 1798, English physician Edward Jenner described a new, safer procedure for protecting against smallpox [1, 2]. Rural residents who got infected by animals which had a smallpox-like disease (cows or horses) were known to have pustular skin lesions on their hands; they suffered a mild infection that left scars phenotypically resembling those after variolation. In addition, people who had contracted cowpox were known to have become immune to smallpox. In 1796, E. Jenner performed the first experiment in which an eight-year-old child was inoculated intradermally with material from a pustule collected from a cowpox-infected woman. To prove that the child had become resistant to smallpox after the infection, Jenner variolated the child after 6 weeks and found that the boy was resistant to this procedure.

Given these findings, to emphasize the protective effect of the used infectious entity against smallpox, Jenner introduced the term “variolae vaccinae” (Latin for cowpox; from Latin vacca (cow)) instead of the term cowpox and called the procedure “vaccine inoculation.” In 1803, Richard Dunning proposed the shortened term “vaccination.” In 1881, at the 7^th^ International Congress of Medicine in London, Louis Pasteur suggested using the term *vaccination *for all protective immunization procedures against any infectious disease [2].

It should be noted that the kingdom of viruses was discovered a century after the introduction of Jennerian vaccination. The first animal virus (the foot and mouth disease virus) was identified only in 1898. The causative agents of smallpox and cowpox proved to be the largest mammalian viruses. Unlike other viruses, their virions, after special staining, had been observed as “elementary particles” under a light microscope as early as 1886; however, the infectious nature of the particles was proven only in 1931 [1].

For many years, the variolae vaccinae virus introduced in vaccination by E. Jenner was believed to originate from the cowpox virus (CPXV) [1, 3]. In 1939, it was found that strains of the virus used for Jennerian vaccination significantly differed in properties from natural CPXV isolates derived from cows [4]. Therefore, they were assigned to a separate species, *Vaccinia virus *(VACV) [1, 3]. The issue of the VACV origin was clarified after the sequencing of the complete genome of a horsepox virus (HSPV) in 2006 [5], which turned out to be closely related to the studied VACV isolates. It is worth noting that E. Jenner considered horses with the pox-like disease to be a source of infection for cows [1-3]. On that basis, it may be assumed that VACV originated from zoonotic HSPV. Apparently, it was HSPV– not CPXV–isolates that were used for Jennerian vaccination in the 19th century. Their descendants were classified as VACV species in the 20th century [6].

It should be noted that, because of lack of knowledge as to the infectious agent nature and mechanisms for protecting a person from smallpox after vaccine inoculation, E. Jenner, in his study published in 1801, predicted that “the annihilation of the smallpox, the most dreadful scourge of the human species, must be the final result of this practice” [1]. Today we know that the etiological agents of smallpox, cowpox, and horsepox are closely related viruses that belong to the genus* Orthopoxvirus *of the *Poxviridae *family. Orthopoxviruses are antigenically close to each other, yield cross serological reactions, and provide an immune defense. The variola virus (VARV) reproduces only in humans, while CPXV, HSPV, and VACV are zoonotic viruses with a wide range of susceptible animals, including humans [3, 6]. Thanks to the international campaign for strict epidemiological surveillance of smallpox, as well as smallpox vaccination carried out under the auspices of the World Health Organization (WHO) since 1958, smallpox was completely eradicated and the last natural case of the disease was encountered in October 1977 [1]. With that, E. Jenner’s intuitive foresight came true. That great achievement of medicine has led to millions of lives being saved.

The eradication of smallpox occurred before the advent of modern practices in virology, immunology, and molecular biology; since there are no animals susceptible to VARV, the development of a protective immune response to smallpox has had to be studied indirectly in surrogate models of smallpox infection. These models include the infection of mice with the mousepox virus (ectromelia, ECTV) or VACV; rabbits with the rabbitpox virus (RPXV) or VACV; monkeys with the monkeypox virus (MPXV), etc. Common patterns of a specific immune response have also been studied using smallpox vaccination of volunteers with VACV [7-9].

Poxviruses are unique among DNA-containing animal viruses, because their entire cycle of reproduction occurs in the cellular cytoplasm in isolated structures called viral factories or virosomes. Brick-shaped virions have rounded faces and are 250–300 × 200 × 250 nm in size. The viral genome of orthopoxviruses is double- stranded linear DNA with covalently closed ends 190–220 kb in size (depending on the species), that encodes about 200 proteins, about half of which are highly conserved and provide the vital function of these viruses [3, 10-13]. The main infectious form of these viruses is the so-called intracellular mature virion (IMV) (*Figure*) that consists of a nucleoprotein core containing the viral genome, a complete transcription system for early viral genes, some other enzymes, lateral protein bodies, and the lipoprotein membrane covering the particle [8, 14, 15]. Mass spectrometry studies demonstrated that VACV IMV includes 85 different viral proteins, with more than 20 of them being associated with the surface membrane [15-18]. A small part of the newly synthesized viral particles are coated with an additional lipoprotein envelope; these extracellular enveloped virions (EEVs) (*Figure*) leave infected cells by exocytosis. EEVs contain an additional eight viral proteins associated with the outer shell [8].

**Figure:**
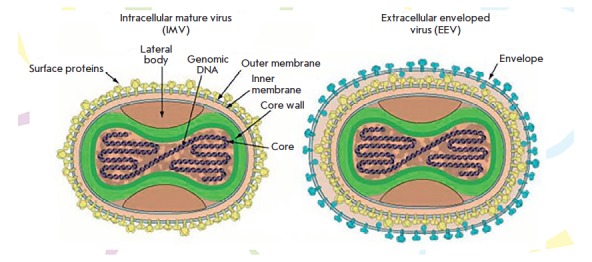
Morphology of intracellular mature (IMV) and extracellular enveloped (EEV) orthopoxvirus virions [14] (ViralZone 2008, with permission from the SIB Swiss Institute of Bioinformatics)

Both live and inactivated VACV vaccines contain mainly IMV particles obtained after the destruction of infected cells and purification of viral preparations. It should be noted that antibodies to both IMV and EEV antigens are induced only when VACV reproduces in the body of an animal. Furthermore, only a live virus in the body of the animal induces the synthesis of protective antibodies to non-virion proteins and stimulates a cellular immune response. That is why inactivated VACV vaccines do not provide complete antiviral protection [8, 19].

## ANTIBODIES SYNTHESIZED IN RESPONSE TO VACV VACCINATION

The antibody response to smallpox vaccination is known to play a crucial role in the protection against a subsequent viral infection [8, 9, 20]. Reliable protection against a smallpox infection was shown to be provided at a virus-neutralizing antibody titer in the blood serum of vaccinated people above 1:32 [21]. In humoral immunity defects, vaccination may not provide smallpox protection. In a B-cell-deficient mouse model, animals were shown not to be able to withstand ECTV re-infection despite noticeable activity of antiviral CD8^+^ T cells [22].

In most cases, VARV and VACV virion proteins have a high identity of amino acid sequences (93–99%) [10, 12], which ensures the high cross-antibody response of these viruses. However, a comprehensive analysis of some individual immunodominant viral proteins revealed differences between these viruses in the profile of induced antibodies. For example, the EEV VACV B5 envelope protein and its homolog, VARV B7, exhibit 23 amino acid differences (93.06% identity) [10], and polyclonal antibodies to VACV B5 cross-react with the VARV B7 homolog. However, out of the 26 monoclonal antibodies to B5, only 16 reacted with the homologous VARV protein in [23].

Huw Davies et al. [24] used microchips with VACV proteins synthesized in a bacterial cell-free system to characterize the profiles of the humoral immune response to vaccination of volunteers with live VACV. The vaccinees were found to develop an antibody response to 47 different viral proteins, with significant individual variations both in the spectrum of antigens and in the production level of antibodies to specific antigens. The results of this and other studies summarized in *Table 1* show numerous viral antigens that often induce a pronounced humoral immune response. This diversity of antigens is believed to indicate redundancy and plasticity of the antibody response in vaccinees, and the presence of antibodies to a large number of antigens creates a “safety network” that provides effective antiviral protection despite individual differences in the spectrum of the produced antibodies [25, 26].

**Table 1:** Main VACV antigens that induced antibody synthesis in more than 25% of vaccinated volunteers [15, 24–26]

Viral antigen^1^	Synthesis time^2^	Function	Localization in virion	Number of tested donors	Antigen-specific antibodies, detection %^3^
A10	L	Structural	Core	73	93.2
H3	L	Structural	IMV membrane	336	90.5
B5	E/L	Structural	EEV envelope	287	88.5
A33	L	Structural	EEV envelope	155	72.9
A27	L	Structural	IMV membrane	336	67.6
A56	E/L	Structural	EEV envelope	155	63.9
WR148^4^	L	Non-structural	Truncated (soluble) ATI protein form	70	62.9
D8	L	Structural	IMV membrane	124	46
D13	L	Non-structural	Enabling IMV assembly	124	46
A13	L	Structural	IMV membrane	123	39
A11	L	Non-structural	Enabling IMV assembly	74	37.8
I1	L	Structural	Core	124	37.1
L1	L	Structural	IMV membrane	205	31.2
A26	L	Structural	IMV membrane	123	29.3
L4	L	Structural	Core	73	28.8
F13	L	Structural	EEV envelope	73	27.4
A14	L	Structural	IMV membrane	124	26.6

^1^Proteins names are given according to the nomenclature of VACV, strain Copenhagen [10].

^2^E/L – early-late, L – late protein production.

^3^Percentage of volunteers with antibodies specific to a given antigen.

^4^Nomenclature of VACV, strain WR. The gene of this protein was deleted in the Copenhagen VACV strain [3].

Antibody biosynthesis is induced primarily in response to virion proteins whose genes are expressed at the late stage of the VACV development cycle (*Table 1*). To date, eight proteins (H3, B5, D8, L1, A17, A27, A28, and A33) have been identified as antigens that induce the production of virus-neutralizing antibodies [8, 25, 27-29]. Involvement of other viral antigens in the development of a protective immune response has not been sufficiently studied. This indicates the incompleteness of our knowledge about the development of a humoral immune response to a smallpox immunization/orthopoxvirus infection.

## VACV-INDUCED CYTOTOXIC T-LYMPHOCYTES

The complex organization of orthopoxviruses is the reason why the mechanism of immune defense against smallpox (and other orthopoxvirus infections) remains not fully understood. Along with the induction of virus- specific antibodies, the response of CD8^+^ cytotoxic T-lymphocytes (CTLs) plays an important role in any control of the infection. A generalized VACV infection (progressive vaccinia) can develop in primary vaccinated people with T-cell immunity defects, while this does not occur in the case of an impaired synthesis of gamma globulins, which indicates the need for a cellular immune response in order to control a primary infection with this virus [8].

As demonstrated in a model of mice pre-infected with an avirulent ECTV strain, antiviral antibodies are necessary and sufficient to prevent the death of animals re-infected with a highly virulent ECTV and the absence of a T-cell immune response does not affect the survival of mice [30]. In mice with B-cell deficiency (antibody synthesis), VACV infection of pre-vaccinated animals was shown to be accompanied by a decrease in body weight, as in unvaccinated mice, but an induction of virus-specific CTLs prevented death and led to a late recovery [20]. This response to re-infection may be explained by the fact that the pre-existing antibodies induced by vaccination can quickly neutralize the infecting virus, while reactivation of the virus-specific T cells generated after vaccination takes several days. Although CD8^+^ T cells are important for circumscribing a primary orthopoxvirus infection, antibodies play a dominant role in the protection against re-infection (infection after vaccination).

In early studies of the CTL response to orthopoxvirus infection/vaccination, researchers focused only on a limited number of antigens. Oseroff et al. [31] performed a bioinformatic sequence analysis of all VACV proteins and pinpointed 6,055 potential peptide T-cell epitopes that were synthesized and used in the analysis of the peripheral blood mononuclear cells of 31 vaccinated volunteers. They identified 48 epitopes from 35 different VACV proteins which effectively interacted with CD8^+^ T-cells of vaccinees. Subsequent studies identified additional orthopoxvirus T antigens [26, 32, 33]. As in the case of antibody biosynthesis, the spectrum of orthopoxvirus antigens inducing a CTL response upon infection/vaccination of humans or animals is characterized by significant individual differences [26, 33]. Viral proteins inducing the most common CD8^+^ T-cell responses in vaccinated individuals are given in *Table 2*. The vast majority of these proteins are synthesized at the early stage of the viral infection; however, a CTL response to some late viral proteins is sometimes detected [26, 33].

**Table 2:** Main VACV antigens that induced production of CD8^+^ T cells in vaccinated volunteers [3, 26, 31–33]

Viral antigen^1^	Synthesis time^2^	Function	Number of tested donors	Percentage of detected antigen- specific T cells^3^
D12	E	Small subunit of the mRNA capping enzyme	81	22.2
C7	E	Inhibition of activity of cellular antiviral factor SAMD9	119	18.5
A47	IE	Unknown	44	18.2
A8	IE	Intermediate transcription factor	68	16.2
O1	IE	Activation of extracellular signal-regulated kinase ERK1/2	75	16.0
J6	E	147 kDa subunit of viral RNA polymerase	80	13.8
D5	E	Nucleoside triphosphatase	154	13.6
M1	E	Ankyrin-like	30	13.3
D1	E	Large subunit of the mRNA-capping enzyme	183	13.1
I8	E	Nucleoside triphosphate phosphohydrolase	70	12.8
C10	E	Blocking of IL-1 receptors	71	12.7
C12	E	Serine protease inhibitor, SPI-1	79	11.4
B6	E	Unknown	45	11.1
B8	E	Secreted γ-IFN-binding protein	120 1	10.8

^1^Proteins names are given according to the nomenclature of VACV, strain Copenhagen [10].

^2^E – early, IE – immediate early protein production.

^3^Percentage of volunteers with CTLs specific to a given antigen.

It is important to note that the immune CD8 response, on the one hand, and the CD4/antibody response, on the other hand, respond to different VACV antigens and involve a broad spectrum of viral proteins [26] (*Tables 1* and *2*). A pattern of the immune responses to orthopoxvirus infection/vaccination with significant personal differences among individuals in the spectrum of antigens inducing an adaptive immune system response was found not only for these viruses, but also for the infectious agents *Plasmodium falciparum *and *Francisella tularensis* [24]. All these facts point to the difficult problem of creating effective inactivated or subunit vaccines for these complex infectious agents.

## PREPARATION OF ATTENUATED SMALLPOX VACCINES

Because mass smallpox vaccination with VACV caused serious side effects, sometimes with lethal outcomes, in a small percentage of cases, the WHO, after the global eradication of smallpox, recommended discontinuation of this vaccination [1, 3]. Because of this eschewing of vaccination against smallpox, most of the human population now lacks specific immunity against not only this disease, but also other zoonotic orthopoxvirus infections [6]. That is why unusually massive outbreaks of orthopoxvirus infections in humans have occurred in various regions throughout the world in recent years [34, 35].

The only effective way to combat the growing threat of orthopoxvirus infections of humans is vaccination [1, 3]. However, the accumulation of immunodeficiency states (HIV infection; patients after organ transplantation; cancer patients, etc.) in recent decades has led to a situation in which mass vaccination of populations with the classic live VACV vaccine is now contraindicated. Therefore, there is an urgent need to develop modern live vaccines that can be much safer compared to the classic smallpox vaccine [36, 37].

The modern approach to virus attenuation involves directed inactivation of virulence genes without affecting the vital virus genes [38]. The virulence genes primarily include genes whose products modulate or suppress the numerous mechanisms of innate and/or adaptive immunity in a virus-infected organism [39]. Orthopoxviruses are characterized by a uniquely large set of such genes. In recent years, many of these genes have been identified, and the properties of the proteins encoded by them have been studied [40]. This diversity of virulence genes, on the one hand, enables the development of different variants of attenuated VACVs and, on the other hand, increases uncertainty in generating the most effective and safe vaccine. Each newly developed VACV variant requires numerous experiments in laboratory animals [9].

VACV attenuation can often lead to a reduced production of the virus *in vivo *and, as a consequence, to a reduction in the induced immune defense of the body. Therefore, an effective antiviral immune response is achieved by introducing significantly higher doses of the newly created virus, compared to the original VACV strains, as well as by revaccination [41]. The absence of knowledge about the functions of and interactions between orthopoxvirus immunomodulatory proteins and the multifactorial mammal immune system results in to a need to use experimenter intuitive assumptions when choosing mutable genes and combinations of them to create new, attenuated VACV strains. The immunogenicity/protectiveness and safety of these strains are tested in various biological systems. Of greatest interest is genomic editing of VACV, which, along with attenuation, can enhance the immunogenicity of the generated virus.

## ENHANCING THE IMMUNOGENICITY OF ATTENUATED SMALLPOX VACCINES

In the course of a long evolution, mammals have developed numerous defense mechanisms against various infectious agents, including viruses. They are divided into non-specific (innate immunity) and specific (adaptive immunity) responses to infection.

Non-specific immediate responses are induced after molecular recognition of conserved microbial components in infected cells and triggering of intracellular signaling cascades that initiate, through the activation of the transcription factors NF-κB and/or IRF3, innate immunity responses [42]. These reactions include cell apoptosis, inflammatory reactions, chemotaxis of macrophages, natural killer cells (NKs), and other cell types to the infection site, complement activation, synthesis of interferons (versatile antiviral proteins), etc. [39, 40]. B cell lymphoma-2-like (Bcl2-like) proteins inhibit/modulate the activation of pro-inflammatory transcription factors and/or apoptosis [40].

The adaptive immune response to infection, which develops over several days, is a complex interaction of various cells which is controlled by cytokines and results in the emergence of B-cells producing specific antiviral antibodies and the generation of virus-specific CTLs. Antibodies interact with viral particles and their components, alone or in combination with the complement, and inactivate them. Cytotoxic CD8^+^ T-cells cause lysis of infected cells [9, 39].

Orthopoxviruses, in the course of co-evolution with sensitive animals, have developed various molecular mechanisms to suppress different stages of innate and adaptive immune response to infection. They encode numerous intracellular proteins that inhibit the development of apoptosis and different stages of molecular signaling pathways that induce the production of interferons, pro-inflammatory cytokines, chemokines, and extracellularly secreted proteins that interact and neutralize the activity of interferons, complement, and various cytokines and chemokines [39, 40]. Usually, these proteins are not vital and have no impact on the efficiency of virus propagation in cell cultures. Targeted inactivation of the genes of these immunomodulatory proteins usually leads to attenuated virulent properties for VACV in an *in vivo *system and, therefore, to its greater safety [38]. However, it may be assumed that removal of the viral genes that suppress the immune response to an infection can increase, in some cases, the immunogenicity of the virus despite a decreased efficiency of virus propagation *in vivo*.

Numerous studies devoted to the deletion (removal) of the genes of VACV immunomodulatory factors have revealed some viral genes whose inactivation, along with attenuation of the virus, increases virus immunogenicity [43-53]. As seen from the data in *Table 3*, these genes include early viral genes whose protein products are involved in the regulation/inhibition of both the innate and adaptive immune response to viral infection.

**Table 3:** VACV genes the removal of which enhances an antiviral immune (protective) response after vaccination

Gene COP/WR/IND^1^	Expression Time^2^	Function	Reference
C6L/022L/D9L	E	Bcl-2-like inhibitor of IRF3 and JAK/STAT activation	[43]
N1L/028L/P1L	E/L	Bcl-2-like inhibitor of apoptosis and NF-κB activation	[44]
K7R/039R/C4R	E	Bcl-2-like inhibitor of NF-κB and IRF3 activation	[45]
A52R/178R/–	E	Bcl-2-like inhibitor of NF-κB activation	[46]
–/013L/D5L	E	Secreted IL-18-binding protein	[47]
B16R/197R/–	E	Secreted IL-1β-binding protein	[48]
A41L/166L/A46L	E/L	Secreted CC chemokine-binding protein	[49]
C3L/025L/D12L	E	Secreted complement-binding protein	[50]
A35R/158R/–	E	MHC Class II Antigen Presentation Inhibitor	[51, 52]
–/169R/–	E	Translation initiation inhibitor	[53]

^1^Genes are designated in accordance with the nomenclature for VACV Copenhagen (COP) and Western Reserve (WR) strains and the VARV India-1967 strain (IND) [3]. A dash denotes the lack of an appropriate gene.

^2^E – early, E/L – early-late transcription.

VACV encodes numerous intracellular Bcl-2-like proteins that inhibit different stages of the signaling cascades of the nuclear transcription factor NF-κB and/or IRF3 activation [40]. Removal of the gene of each of the four proteins from this family (C6, N1, K7, and A52) was shown to lead to increased production of NK cells, enhanced CD8^+^ T-cell immune response to VACV infection, and an increased protective effect (protectiveness) against re-infection [43-46].

Inflammatory processes play an important role in early non-specific protection of the organism against a viral infection. They develop rapidly to limit virus dissemination within the first hours and days that follow an infection, while the adaptive immune response is being erected. Cytokines, such as IL-1β, IL-18, TNF, and γ-interferon, which trigger molecular inflammatory cascades in a particular chemokine expression, are known to play the key role in the induction of inflammatory reactions. Chemokines are chemoattractant cytokines that regulate the migration and effector functions of leukocytes, which play an important role in inflammatory response development and protection against pathogens. The complement system consists of more than 20 plasma proteins. The antiviral action mechanisms of the complement system include neutralization of the virus, lysis of virus-infected cells, and enhancement of the inflammatory and adaptive immune response [40]. A deletion of individual genes encoding IL-1β, IL-18, chemokine, and complement inhibitors was found not only to decrease the virulent properties of VACV, but also to enhance the immunogenic properties of this virus [46-49] (*Table 3*).

The early intracellular VACV protein A35 was shown to inhibit presentation of viral antigens by the major histocompatibility complex of class II [54, 55]. Removal of the *A35R *gene increases the production of virus-specific antibodies and enhances the protectiveness of VACV [51, 52].

Investigation of the function of the *169R *gene in the VACV WR strain revealed that the protein encoded by the gene inhibits the initiation of mRNA translation in an infected cell, without affecting viral mRNA translation in isolated virosomes. This underlies the wide range of the effects of protein 169, in particular the inhibition of the innate immune response to a viral infection [53]. Deletion of the *169R *gene resulted in enhanced production of pro-inflammatory cytokines and chemokines, increased lung infiltration by leukocytes, and, as a result, a stricter CD8^+^ T-cell immune response and more effective antiviral protection against repeated infection upon intranasal infection of mice with a mutant VACV.

Smallpox survivors are known to acquire lifelong immunity against the disease. VACV vaccination provided effective protection against this especially dangerous infection; however, re-vaccination was required to maintain a reliable level of protection against smallpox for a long period of time [1]. In this regard, it is noteworthy that the causative smallpox agent, VARV, lacks at least four genes in its genome, the removal of which enhances the antiviral immune response, which involves various molecular mechanisms (*Table 3*).

There have been more or less successful attempts to obtain attenuated and highly immunogenic VACV strains using targeted inactivation of several viral genes.

In the NYVAC strain, a simultaneous deletion of three genes of Bcl-2-like proteins (*A52R*, *B15R*, and* K7R*) was shown to enhance the innate immune response in infected mice, which resulted in increased chemokine production and greater migration of neutrophils, NK cells, and dendritic cells into the infection site [46].

Genes encoding IL-18-binding (*C12L*), IL-1β- binding (*B16R*), and CC-chemokine-binding (*A41L*) proteins, as well as a Bcl-2-like protein (*A46R*), were deleted from the VACV MVA strain genome [56]. The produced VACV variant with four deleted immunomodulatory genes resulted in a higher level of antiviral antibodies in rhesus monkeys compared to the initial VACV MVA.

A higher adaptive T-cell immune response was induced by a VACV MVA strain with intentionally deleted three genes encoding an IL-18 binding protein (*C12L *or *013L *for VACV WR), a Bcl-2-like protein (*A46R*), and 3β-hydroxysteroid dehydrogenase (*A44L*) [57].

The VACV LIVP strain was used to create a recombinant variant with five impaired virulence genes encoding hemagglutinin (*A56R*), a gamma-interferon- binding protein (*B8R*), thymidine kinase (*J2R*), a complement-binding protein (*C3L*), and a Bcl-2-like apoptosis inhibitor (*N1L*). Inactivation of these virulence genes was shown not to affect the reproductive properties of VACV in mammalian cell cultures. The produced VACV strain was characterized by significantly lower reactogenicity and neurovirulence compared to those of the original LIVP. Upon subcutaneous administration to mice, the recombinant VACV variant induced the production of VACV-neutralizing antibodies at a level comparable to that of the parental LIVP strain [38]. To increase the production of virus-specific antibodies, the *A35R *gene was additionally inactivated (*Table 3*). The produced LIVPΔ6 strain induced a significantly higher level of virus-neutralizing antibodies in mice and provided greater protection than the original VACV strain [52].

Given the fact that removal of individual genes of the VACV Bcl-2-like proteins N1, C6, or K7 not only led to an attenuation of the virus but also increased its immunogenicity [43-45], a VACV WR variant lacking these three genes was created. The obtained triple VACV mutant did not lose its ability to efficiently propagate in cell culture, but in the *in vivo *system it was more attenuated compared to mutants with single deletions of these genes and caused a decreased production of virus-neutralizing antibodies and specific CD8^+^ T cells [58].

Summarizing the results of these studies, it may be concluded that the development of safe and highly immunogenic VACV variants should rest on a balance between attenuation and immunogenicity. Since our current level of knowledge does not allow us to predict the results that might be achieved by the impairment of several target VACV genes, each produced virus variant should be carefully studied in different model animals.

## Abbreviations

WHO: World Health Organization
CPXV: cowpox virus
CTL: cytotoxic T lymphocyte
EEV: extracellular enveloped virion
HSPV: horsepox virus
IMV: intracellular mature virion
NK: natural killer
VACV: vaccinia virus
VARV: variola virus
